# Regaining creativity in science: insights from conversation

**DOI:** 10.1098/rsos.230134

**Published:** 2023-05-17

**Authors:** Ruth M. Morgan, Roger L. Kneebone, Nicholas D. Pyenson, Sabrina B. Sholts, Will Houstoun, Benjamin Butler, Kevin Chesters

**Affiliations:** ^1^ Department of Security and Crime Science and UCL Centre for the Forensic Sciences, University College London, 35 Tavistock Square, London, UK; ^2^ The Arista Institute, UCL Engineering Front Building, Torrington Place, London WC1E 7JE, UK; ^3^ Centre for Engagement and Simulation Science, Department of Surgery and Cancer, Imperial College London, London, UK; ^4^ Royal College of Music–Imperial Centre for Performance Science, Royal College of Music, London, UK; ^5^ Department of Paleobiology, National Museum of Natural History, Washington, DC, USA; ^6^ Department of Paleontology and Geology, Burke Museum of Natural History and Culture, Seattle, WA, USA; ^7^ Department of Anthropology, National Museum of Natural History, Smithsonian Institution, Washington, DC, USA; ^8^ Horasis, Maurerstrasse 2, 8500 Frauenfeld, Switzerland; ^9^ Harbour Collective, LABS House, 15–19 Bloomsbury Way, Holborn, London WC1A 2TH, UK

**Keywords:** creativity, science leadership, conversation, interdisciplinary, diversity

## Abstract

The ‘early modern’ (Renaissance) workshop was predicated on the idea that informal, open-ended cooperation enables participants to experience difference and develop new insights, which can lead to new ways of thinking and doing. This paper presents the insights that emerged from a conversation event that brought wide-ranging voices together from different domains in science, and across the arts and industry, to consider science leadership as we look to the future in a time of interlocking crises. The core theme identified was a need to regain creativity in science; in the methods of scientific endeavours, in the way science is produced and communicated, and in how science is experienced in society. Three key challenges for re-establishing a culture of creativity in science emerged: (i) how scientists communicate what science is and what it is for, (ii) what scientists value, and (iii) how scientists create and co-create science with and for society. Furthermore, the value of open-ended and ongoing conversation between different perspectives as a means of achieving this culture was identified and demonstrated.

## 1. Introduction

Science has traditionally been highly creative. Observations have led to theories in every domain of the natural world and generated new discoveries. Creativity, the use of ideas and the imagination, is at the heart of this. The demonstration of molecular chirality by Pasteur in 1847 was ground-breaking science, and arguably in part a result of his experiences as an artist using lithography [1,2]. Yet in more recent times and in many areas and sectors, science is increasingly operating within a post-industrial revolution production model with a rational economic foundation [3] that seeks, values and rewards narrowly defined (often quantitative) measures of productivity and excellence [4].

The significant challenges we face globally are shaped by conditions of volatility, uncertainty, complexity and ambiguity [5–7]. Holistic approaches will be critical to identifying and anticipating these challenges, making breakthroughs and finding solutions [8]. To achieve this, however, science needs to rediscover its creative roots, or risk becoming a tool that is only looked to in order to answer specific, often analytical, questions. There is of course deep value in addressing such analytical questions; these will remain, and answering them, is foundationally important. However, there is also a need to identify solutions that can emerge from a reframing of existing approaches or understanding of a system, or the synthesis that can occur at the intersections of disciplines. For example, the discovery of the antimalarial drug artemisinin (ginghaosu) by Tu and her team in 1972 [9] derived from a deep knowledge of ancient Chinese herbal remedies rather than a medical degree. Creativity, therefore, is a critical characteristic of science and how science is carried out.

It is also important to recognize that science cannot be separated from scientists, the people who carry it out in communities, both with and for society. To maintain and grow creativity in scientific endeavours it is more important than ever that scientists are encouraged and enabled to be creative; for that creativity to be valued in both pure and applied fields; and for multidisciplinary and interdisciplinary settings to be created and sustained. As scientists we need to communicate the value of science that has creativity ‘woven into its fabric’ and demonstrate the necessity of creativity across the board as we collaborate across disciplines (science, social science, engineering, arts and humanities) and industry to generate solutions to the questions that will shape the future of our species and planet.

This paper presents insights that emerged during a curated conversational event held in London, UK, in March 2020, addressing global science leadership for the future. The conversation was designed to create space for a group of participants from different backgrounds to engage as equals in an informal and open-ended dialogue, and to arrive at new insights. There were nine participants^1^ and the group included international scientists from different disciplinary domains, a clinician, a futurist, a strategy expert in advertising, a science publisher and a professional close-up magician with a background in mechanical engineering. Participants were from the UK, USA and Asia, and represented early, mid and senior career stages. A conventional approach might have been to bring only senior subject specialists (in this case scientists) together to work through an agenda and reach a set of outcomes in a format agreed in advance of the meeting. Instead, we used a conversational approach to explore the topic in an alternative way, bringing different perspectives in terms of sector, seniority, experience and geography. The outcomes of the conversation were a product of the participants' experience and skills, as well as the listening, reflecting and sharing that took place. These outcomes would not be replicated on another day or with different participants (as might be more likely in a more traditional session). A conversational approach, with its less rigid structure, offers the potential to generate new ideas, identify synergies in contrasting fields and sectors, and articulate important issues to frame future conversations.

This conversation took place on the day that the World Health Organisation declared that COVID-19 was a pandemic. This pandemic went on to affect every society across the world, and during that time the roles of science and scientists in society became increasingly scrutinized.

This article synthesizes ideas that emerged from the conversation event, along with subsequent reflections made possible by the restrictions on events during the early pandemic in many parts of the world. Firstly, we consider the context of science, how it is perceived by different stakeholders and what these different stakeholders consider its purpose to be. Secondly, and against that backdrop, we explore why creativity is so important for the production and application of science when tackling complex problems, and what is needed to achieve creativity in science. Thirdly, we highlight challenges and potential barriers to creating a culture of creativity in science, drawing on insights that materialized from different perspectives captured through this conversation on global science leadership.

## 2. What is science considered to be ‘for’?

Science is a broad term^2^, yet it is generally considered to encompass the systematic study of the world by observation and experiment to seek and apply knowledge based on evidence. Science has many stakeholders, spanning different sectors and areas of activity. Within this knowledge ‘ecosystem’ there are diverse views of the purpose of science (what science is ‘for’), and what value it has (table 1).

**Table 1.** Themes that emerged from the conversation addressing what science can be considered to *be* and to be *for* from different stakeholder perspectives.

sector/stakeholder	context	what the contribution and/or value of science is	key challenges encountered
government and policy	governments operate at national and international levels taking a strategic overview of key challenges and the intersecting issues that contribute to those challenges. In democratic settings, governments and policy tend to operate broadly within political timeframes (often 2–5 years, in alignment with political elections) when identifying priorities and developing strategy [11]	science is often a tool that is deployed to find or create solutions to challenges prioritized by government agendas and often in the form of products or approaches to address a specific challenge. It can also be used to justify political decisions	while science and technology is often seen as a key contributor to innovations and offering solutions to challenges, there can be challenges in aligning different policy areas to accomplish the desired outcomes. It can also be challenging to bring together multiple stakeholders to achieve inter-organizational ideational alignment and co-evolution [12]
			a tendency to present preferred solutions for a particular challenge to the science community, with science expected to fulfil that need with a product or definitive solutions [13] in predetermined timeframes
business	generally, business seeks to create value for stakeholders and/or shareholders through the production of products or services. Businesses develop in-depth knowledge of a specific sector across local/regional/national and disciplinary boundaries to be aware of needs in societies, and service those markets by developing the capabilities to address those needs with solutions	science is a tool for innovation either in production processes, sustainability [14] or products that provide solutions for customers [15]	the evaluation of success is often in financial terms (such as benefitting shareholders), and often considered over shorter timeframes (such as fiscal years)
education	education can take many forms. It is generally focused upon ensuring that, as a society, we have a knowledge of our world, and the tools we need for society to work well and be productive (often with a focus on economic prosperity)	science is often framed as a set of principles and methods to be learnt and reproduced [16], rather than as an interaction and a way of thinking to embrace, apply and experience [17]	there can be a focus on understanding mechanisms within a current paradigm to achieve qualifications, rather than valuing and developing a mindset for exploring how to identify patterns, synthesize, falsify and develop new ideas or reimagine how things work
research	research seeks to generate new knowledge about the physical, virtual and human world. Funded research within the higher education system is often broadly framed by national and international funders and their assessment of key fields, challenge areas, and attitude towards risk. This may be framed by financial and/or political timeframes [12]	science is a means of contributing to the body of knowledge, addressing gaps in the knowledge base and achieving solutions to challenges or problems that exist at a range of scales	science research is often proposed, crafted, carried out and its outcomes delivered in response to calls from funders. These calls are shaped by what government(s) and industry perceive to be valuable. This can create tension between ‘pure’ and ‘applied’ science and mission or curiosity driven research. The problem is exacerbated by funders needing to demonstrate ‘value for money’ in short timeframes
			in recent years funders have required that funded science research should demonstrate ‘impact’ [18,19]. This is predominantly measured in terms of citations, financial outcomes such as spin out companies and new products that contribute to national gross domestic product, or the creation of a specific piece of policy [20–22]
			an increased focus on minimizing risk reduces opportunities for ‘blue skies’ exploratory research and innovation approaches [23]
society	societies are made up of individuals who individually and collectively perceive the world through many different lenses. The public are key actors in setting out cultural norms, institutions and governance structures	science (and the scientific method) is often perceived to be logical, sequential, mechanistic and an unequivocal source of certainty, and a source of solutions to big challenges (such as a global pandemic) in terms of both managing them and creating ‘cures’. It can also be considered a historical colonial enterprise that does not serve or include all people and all societies with equity (e.g. [24])	there is often a tension between so called pure/basic and applied research. Laboratory settings can create environments where variables can be controlled and causation between variables tentatively established, but often demonstrating impact in societal terms can be challenging. By contrast scaling these insights and outcomes to complex ‘real world’ scenarios characterized by variability and uncertainty can have clearer impacts but it can be challenging to communicate and gain acceptance of the necessary provisionality of science findings, theories and processes

Across different sectors science is embedded in society to greater and lesser extents, in both explicit and tacit ways. Science can be seen as a tool to address a specific challenge and generate a solution. In this setting, science is looked to when a problem arises. Framed in this way, science can assist with articulating the problem at hand. It can then be deployed to find answers, often through innovation. For example, in an acute public health crisis such as a pandemic, science can offer insights into the ‘problem’ (such as the causal pathogen, its transmission, rates of infection, symptoms in the population, infrastructural capacity within the healthcare system) and be deployed to drive the delivery of solutions (such as effective vaccines and rapid tests that can be produced at scale in appropriate timeframes, equipment to reduce transmission, and healthcare pathways to mitigate the impact on patients). In a business setting, viewing science as a tool can result in innovative solutions to perceived needs, and marketable products that can generate revenue (such as the kind of telecommunication products that became essential for social and professional interactions under COVID-19 lockdown restrictions).

In other settings, science is considered to be a means of producing knowledge that can assist with solving questions in different domains. At differing stages within the criminal justice system, for example, science is enlisted to assist with reconstructing crime events and offering insights into who may assist an investigation, or what can be presented in court as evidence [25]. In marketing and advertising, science can be used to persuade an audience of the value or efficacy of a particular product [26]. Scientific approaches are increasingly used as a means for understanding a particular audience, exploring what they care about and how they make choices, particularly with increasing access to ‘big data’ [27], in order to craft a campaign and its messaging in a way that will resonate with the intended audience. In the domain of magic, there is a growing consideration of the ‘science of magic’. Advocates outline the value of utilizing a systematic method of investigation established in science to consider the nature of the experience created by magic, how individual ‘tricks’ create this experience, and how the knowledge base in magic can be collated systematically and comprehensively [28] to produce knowledge.

Challenges can arise from different views of what science is and what it is for. For example, science in the criminal justice system has been robustly critiqued in terms of how evaluative opinions about the meaning of a piece of scientific evidence in a case are framed and presented [29,30]. This has arisen, in part, because the justice system looks to science to provide clear and definitive answers to pertinent questions that can be considered by a court to be beyond reasonable doubt. In broader societal settings too, science is often perceived as being an orderly, logical, methodical approach that offers clarity. When science is used in advertising or policy making to back up a specific understanding of a situation or messages about an issue or a product, inherent limitations or levels of uncertainty are not clearly incorporated into that messaging [31]. In some contexts, the attributes of variability, uncertainty and the evolution of understanding that are all intrinsic to science in its broadest terms are neglected in favour of a perception of science that offers more definitive and clear insights. The inescapable provisionality of scientific knowledge often goes unrecognized.

The diversity of views that becomes apparent when the questions of ‘what is science?’ and ‘what is it for?’ are considered from different vantage points reflects the nature of science itself. Though scientific work is logic and method driven, scientific knowledge is constantly evolving, dynamic and in a state of flux. Though science has the potential to address a broad range of questions, and to offer insights of value in contrasting settings, the expectations of different stakeholders and different contexts can be at odds.

For science to continue to bring value, deepen understanding and make progress across a broad range of contexts and challenge areas, it is possible to argue that it needs to be seen by its stakeholders as broad and creative, combining logical analysis with agile and collaborative thinking. At its most effective, science presents observations that are context specific and generalizable, offering transparency regarding uncertainty and limitations that can inform and assist those charged with making decisions. It offers an opportunity to generate deeper knowledge and understanding in a specific area of focus, brings diverse perspectives to reconsider and re-imagine established ideas, finds synergies from other domains and sectors, and make breakthroughs to deliver innovation in different contexts.

## 3. Creativity and science

### 3.1. Why do we need creativity in science?

To tackle the big questions and challenges in the world, collective approaches that combine an understanding of broad systems with a diverse range of perspectives, approaches and tools can generate outcomes that move us forward. For example, ‘collective intelligence’ that draws diverse groups together has been shown to increase innovation, productivity, the generation of new ideas and mobilization of applications [32]. Bringing such diverse insights together is important, because it is often when those insights are considered in novel contexts that leaps forward happen [33].

Creativity is key to successful collaborations, underpinning the innovative thinking that reconsiders and rearranges information to generate novel and useful solutions or products [34–36]. Creative thinking, critical questioning and re-imagining the status quo are central to scientific enquiry that may find solutions to global challenges while driving innovation and shaping the future [33,37]. However, creativity is often seen as the preserve of the arts and craft domains [38]. We believe this is a fundamental and dangerous misconception. If science is to address the global challenges we face—and be part of the conversation about what kind of world we want to live in—it must have creativity woven into its fabric, identity, methods and vision. This requires us to open the conversation to people who think in different ways, embracing human qualities of being a scientist (such as the ability to perform, persuade and communicate) alongside abstracted findings and depersonalized discoveries.

### 3.2. How do we achieve creativity?

It has been argued that creativity cannot be achieved through a planned process with a pre-defined goal [39]. Rather creativity and originality emerge where there is openness to new ways of thinking and doing [40,41]. Creativity depends on individuals and the environments in which those individuals work. ‘Environments’ encompass physical and virtual space, together with the individuals that form the human community within each setting [42,43].

Creativity is an individual quality (often characterized as a ‘lightbulb moment’) [44,45] and a social phenomenon where creative insight is reached through interaction with a group [46]. To achieve creativity in science, whether individually or collectively, we need to balance how scientists think and carry out their science [33] with the environments and infrastructures in which they work [38,47]. Team autonomy and personal responsibility along with meaningful social interactions can encourage creativity [42]. This is especially important if creativity is to become a reliable component of scientific work, rather than occasionally appearing as the proverbial lightning strike.

Many models of creativity have roots in the work of De Bono and the framework of lateral and vertical thinking [33]. Lateral thinking (the process of using information to achieve creativity) results in the generation of new ideas and rearrangement of existing insights to challenge existing paradigms and create new ones. It seeks out extraneous information, challenges established premises and values ‘being wrong’ as a prompt to generate new ideas. By contrast, vertical thinking (traditionally thought to be dominant in science work) is more conventional. Here, a conclusion is reached through a sequence of logical steps, each being rigorously justified. Yet lateral thinking is not a substitute for vertical thinking—both are necessary, and their combination is cumulative and complementary. While lateral thinking is generative, vertical thinking is selective. Vertical thinking is concerned with proving or developing observable patterns and testing hypotheses, while lateral thinking restructures patterns (insight) and provokes new ones (creativity).

While the way an individual thinks and carries out their work is an important element of achieving creativity, the environment within which that individual operates is also critical. Creative knowledge environments [47] exert a positive influence on those engaged in creative work, whether individually or collectively, or in a single institution or collaboratively [38]. Environments that foster creativity and encourage creative approaches provide autonomy or freedom for individuals or groups of individuals, while ensuring that appropriate resources are available [48]. In the realm of science, there is value in building space for serendipity—chance encounters and opportunities for conversations that bridge disciplines, industries and national borders [38].

Much scientific work takes place in institutions with well-established infrastructures and methods of knowledge creation, with specialist languages and modes of communication and dissemination. Now it is important to consider our existing infrastructure and commit to learning (and unlearning) how we can bring creativity into the fabric of what science is and how science is woven into society.

## 4. Challenges for creating a culture of creativity in science

Hosting a conversation to consider the future of global science leadership was an experimental step, designed to create the opportunities and space for lateral thinking and critical questioning. The event brought together people from a range of different sectors (science, publishing, the arts, advertising and consultancy), and scientific fields (forensic science, anthropology, geology, data science, psychology, security science) in a spirit of exploration without preconceived notions of outcome or result.

A successful conversation is dynamic, responsive, and diverse. Fruitful conversations share characteristics of the ‘early modern’ (Renaissance) workshop, as summarized by Sennett [49]. Predicated on the idea that informal, generous-minded cooperation is the best way to experience difference and develop new insights, informal settings offer opportunities for individuals with different backgrounds and experiences to connect open-ended interaction [17,49]. This removes the need to know the end result at the outset and frees those in the conversation to explore ideas and viewpoints in a dynamic and responsive way. Cooperation holds everything together, with each contributor seeking to gain through exchange rather than the purpose being for one party to gain at the expense of the others [49]. Indeed, it is often in long standing cooperation that breakthroughs are made [50].

Three interrelated themes emerged from this conversation event: (i) how we communicate what science is and what it is for, (ii) what we value, (iii) how we create and co-create science.

### 4.1. How we communicate what science is and what it is for

The term ‘identity’ prompts debate around how diversity (framed widely) encompasses individual and collective entities, disciplines and institutions [51–53]. Questioning and exploring the identity of science forces us to consider the nature and purpose of scientific work [30]. That identity is produced through social interaction and relationships that evolve and are expressed in different ways according to time and place. How and what we communicate of that identity will play a significant role in what science will become [16,54].

A key theme from the conversation was that of multiple audiences. Communicating is a two-way process, involving both scientists and ‘publics’. Determining what science *is* and what it is *for* to those different audiences is a challenge with far reaching consequences. Mass media plays a major role in shaping which stories are told, how those stories are crafted, how different audiences experience those narratives, and ultimately how science is woven into society and culture. Important questions emerge about the stories that science tells and that are told about science, and the narratives that reach different audiences, whether the general public, industry, government or other scientists. Additional questions arise in terms of who should be telling those stories, and the implications for the role of educational settings in nurturing the next generation of scientists. For example, the work of TAIK that seeks to inspire, inform and educate in order to change a specific narrative [55] illustrates the importance of storytelling and the storytellers. If science is to be a part of global, national and local agendas rather than a tool brought in to find a solution to a specific problem, we must recognize how these narratives resonate with different audiences.

### 4.2. The value of science

#### 4.2.1. How science is valued

Our values set priorities and agendas, shaping strategic goals in industry, business, education, governance and research settings. The social contract for science [56,57] was predicated on a partnership between government (financially supporting universities in carrying out basic research), the scientists in receipt of that funding (expected to behave with integrity and professionalism), and the delivery of research outputs (expected to confer wider benefit to society). This framework relied on delegating science and its advancements to the scientific community and promoted a model where pure research and basic science transitioned to applied research which created products and services that benefit society.

In recent times there has been a shift in focus towards productivity, so that research is not only funded largely by the state, but its methods and outputs are monitored, and science is incentivized for (economic) productivity [58]. This shift has driven a move toward economic consideration of the value of science, underpinned by a business framework (seeing value as equating to benefits achieved after effort, risk and price are accounted for) [59]. This raises challenges since economic assessment does not always equate to perceived value. This runs the risk of restricting future scientific development to a system that only values financial gain, rather than incorporating a spectrum of more broadly beneficial values.

Some areas of science can perform well within this framework, often through the creation of intellectual property that leads to marketable products and spin-out companies. Yet many areas of science cannot be evaluated in financial terms. For example, some outcomes create better systems and structures, which contributes to societal wellbeing. These outcomes often emerge when creativity and exploration have played a central role in articulating a research question or re-considering the methods which impact upon the real world (for example, the field of behavioural economics). Sometimes such outputs indirectly lead to the creation of financial rewards (greater productivity, reduced healthcare costs, or creating cultures that attract highly productive individuals and communities across the sciences, arts, industry, business, government landscapes). In other instances, however, science produces outcomes that benefit and enrich society in less obvious ways which may not be identified in shorter timeframes [60]. Capturing the value of such outputs in terms that have traction within existing mindsets and institutional infrastructures has remained elusive. Developing that traction is key to demonstrating the value of creativity in science, and only then can we build a culture in science and the environments that foster and enable creativity.

#### 4.2.2. What is valued in science

One challenge is to articulate what forms of productivity and output are valued as this continues to affect which skills and attributes of scientists are valued and rewarded [61]. Part of the drive to evaluate science and demonstrate productivity is to demonstrate its value to society. Yet increasingly we are encountering challenges where traditional approaches cannot solve long-standing, so-called wicked problems, whether in the environment, health, education or justice domains. All these challenges are complex, interconnected and interrelated. Interdisciplinary approaches that synthesize knowledge and promote lateral thinking are a key to achieving solutions [62]. Yet traditional interdisciplinary approaches have not provided solutions either [25,63]. To reap the benefits of true interdisciplinarity, science must span a spectrum from pure or basic science to translated and applied science, incorporate the wisdom from social science, humanities and the arts, as well as supporting communities that incorporate both specialist experts and expert generalists within and beyond traditional disciplinary categories [60,64].

An observation that emerged from the conversation was that expert generalists have a form of expertise that is difficult to articulate and frame within existing institutional infrastructures [65]. Expert generalists are lateral thinkers who can identify patterns, connect dots across different topics, and improvise. They operate at the intersections of disciplines, and often at the boundaries of science and its stakeholders, rather than developing focused expertise in a more clearly defined field. As a result, expert generalists have traditionally faced challenges to demonstrate their value and credibility [66] in systems that recognize and reward specialist knowledge. There are encouraging signs that this is changing [64,67] and a growing recognition that both types of scientist [68] are key to science that can serve society and our planet.

### 4.3. How we create and co-create science

Acknowledging the importance of creativity opens opportunities for science to become a means of innovation at a range of scales and time frames. When science is used as a tool to deliver a product for a specific challenge, that challenge is often acute (or soon to become so) and articulated in specific terms with a narrow focus. Such science involves transferring information to a specific audience, or the creation of a tool that fixes the problem at hand. It can be perceived as something that is done *to* people. If we create an experience of science for different audiences that is woven into the everyday, science becomes something that is done *with* people and ultimately *for* people [69]. Achieving this requires creativity—a lens through which to recast the strength and perceived quality of science.

Science that is done for society must be intrinsically connected to that society, highlighting the importance of co-creation and co-ownership. A growing body of science is co-produced by scientists working together with so-called ‘lay authors’ by incorporating different approaches and ways of seeing [70,71]. Science undertaken in this way (including crowd science) can become more creative, diverse and have more impact [72]. A flat hierarchy and accessible context for those interactions are essential for this kind of co-creation, in which parties bring different, yet essential, perspectives that includes voices that have not been included in the past.

Making the step from science insights to real world impact is often a challenge, and one that is becoming more widely acknowledged as metrics of research excellence incorporate measures of research impact [20,73–77]. The advertising industry is often credited with seeking to take the amazing and making it hypnotic. Advertising, at its most powerful, creates stories that resonate with a specific audience, in a particular time and/or place, for a defined purpose or call to action—thereby creating a recognizable brand. Traditionally, science and the scientific community have not been comfortable with the idea that science is a brand. However, if the aim of science is to have impact, incorporating attributes of storytelling, rhetoric and communication honed in the advertising world could be used in powerful ways.

Here the idea of the conversation can be valuable. Effective storytelling happens when the storyteller knows the intended ‘audience’ well [78]. Conversation is one way of getting to know an ‘audience’, in a way that is ongoing, reciprocal and open-ended. Scientists and those in the audience are agents of change. Ongoing dialogue offers one way of ensuring that the best questions are asked, and that the insights from science are presented and communicated compellingly [61].

## 5. Conclusion

This conversation event that addressed science leadership for the future brought a diverse group of voices together and created two important opportunities. Firstly, it highlighted the value of creating space for exploring ideas in a free-flowing setting. Secondly, it showed the power of open-ended informal, cooperative dialogue sharing radically different perspectives. This context provided licence to explore different ways of ‘seeing’ and to rearrange ideas and frameworks.

Science that has creativity woven into its fabric, which is sensitively infused into education, policy and governance, industry and business, has the potential to catalyse and foster a culture of innovation, problem solving, diversity and anticipation. This requires a continuing dialogue within the science community to nurture and grow creativity in the way science is carried out, communicated and embedded within society. There also needs to be conversation beyond the academy, incorporating wider perspectives and key decision-making bodies in society (government, parliament, business, industry, non-governmental organizations, charities and education sectors). As this conversation evolves and grows over time, a collective experience of science will permeate strategic decision-making and the articulation of vision and priorities.

Ultimately science must re-engage with creativity and re-capture its creative roots. We need to create opportunities to imagine and ask, ‘what if?’ [79]. We need to consider what works well within the status quo but also take opportunities to explore and consider additional goals and pathways. This requires a collective approach that transcends disciplinary, institutional and international boundaries. It will require investment in creating physical and conceptual spaces where lateral thinking can thrive, and traditional framings of excellence re-visited. It will also require commitment to building bridges within and beyond the academy, communicating science in engaging and effective ways, and investing in the next generation of scientists so that they have the skills and networks to be the voice of science.

Science is more than a tool to be deployed when a problem arises, and more than an experiment confined to a specific laboratory. In the same way, medicine is not simply applied biology, and magic is far more than a technique (but the use of knowledge and insights to create an experience with an audience). Science is a way of thinking and doing, a means of testing and evaluating, an approach to deliver breakthroughs and stepwise change. If we can weave creativity into all aspects of scientific work, we will establish pathways that enable innovative thinking and science to be a part of a collaborative approach to finding solutions to the long-standing global challenges.

## Data Availability

This article has no additional data.

## Footnotes

1 The group included three women and six men and none of the participants were renumerated for their time. Participants were invited to join a conversation event and each received a one page overview that set out the topic (science leadership) and its timeliness as well as an overview of the conversation approach. The conversation was facilitated by Prof. Roger Kneebone and Prof. Ruth Morgan, and an audio recording was taken (with permission).

2 In this paper the focus is on a scientific (or Western) model of knowledge, which is generally hypothesis driven, as opposed to Indigenous or Traditional knowledge systems that are culturally rooted and transmitted [10].
